# A novel drug that reduces pneumococcal toxicity by targeting pneumolysin (PLY): efficacy of the traditional Chinese medicine *Radix Paeoniae Alba*

**DOI:** 10.3389/fphar.2025.1609457

**Published:** 2025-11-10

**Authors:** Yan Xu, Jing Han, Yonglin Zhou, Liping Sun

**Affiliations:** 1 Anhui Huatuo Academy of Traditional Chinese Medicine, Bozhou Vocational and Technical College, Bozhou, Anhui, China; 2 Affiliated Hospital of Changchun University of Chinese Medicine, Changchun, Jilin, China; 3 Changchun University of Chinese Medicine, Changchun, Jilin, China; 4 School of Life Sciences, Ningxia University, Yinchuan, Ningxia, China

**Keywords:** Radix Paeoniae Alba, *Streptococcus* pneumoniae, pneumonia, pneumolysin, traditional Chinese medicine

## Abstract

**Objective:**

While existing virulence-targeting strategies predominantly rely on single-component inhibitors that exert evolutionary pressure, this study pioneers an innovative approach using the multicomponent traditional Chinese medicine *Radix Paeoniae Alba* (RPA). Unlike conventional monotherapeutic agents, RPA uniquely inhibits pneumolysin (PLY) oligomerization without affecting bacterial growth, thereby circumventing resistance development—a critical limitation of current therapies. We aimed to elucidate the novel mechanism by which RPA attenuates *Streptococcus pneumoniae* pathogenicity by inhibiting the pore-forming activity of PLY while preserving host microbiota homeostasis according to the holistic TCM philosophy.

**Methods:**

Using a murine pneumococcal infection model, we evaluated the therapeutic effects of RPA on lung inflammation. Hemolysis assays and A549 cell viability tests were performed to assess PLY inhibition. Western blotting was used to characterize the PLY oligomerization dynamics following RPA treatment. Bacterial growth curves confirmed the nonantibacterial nature of RPA.

**Results:**

RPA significantly reduced pulmonary inflammation (p < 0.05) and the levels of inflammatory cytokines (IL-1β, IL-6, and TNF-α) without altering *S. pneumoniae* growth. Mechanistically, RPA inhibited PLY oligomerization (64 μg/mL) in a dose-dependent manner, thereby preventing erythrocyte hemolysis and alveolar epithelial damage.

**Conclusion:**

This study provides the first evidence that a multicomponent TCM achieves targeted antivirulence effects by blocking PLY oligomerization, providing a resistance-proof therapeutic strategy. Our findings bridge the “body-strengthening and evil-eliminating” principle of TCM with molecular pathogenesis, highlighting the potential of RPA as a novel antivirulence agent for pneumococcal infections.

## 1 Introduction

*Streptococcus pneumoniae* poses a serious global health threat (Michaud et al., 2001), with its impact potentially exceeding that of cancer, diabetes, and AIDS (Mizgerd, 2006). As a primary cause of bacterial pneumonia (accounting for approximately 35% of cases), *S. pneumoniae* is the leading cause of community-acquired pneumonia (CAP) worldwide and a major cause of death in children under 5 years old (Meyer, 2024). The World Health Organization (WHO) reported that approximately 1.6 million children die annually from pneumococcal infections, mostly infants younger than 2 years old (Ceyhan et al., 2018). In developing countries, the incidence and mortality of pediatric pneumococcal pneumonia are particularly high. Infected children often present with high fever, cough, and shortness of breath, which can progress to life-threatening complications such as respiratory or heart failure (Li et al., 2016). Therefore, effective prevention and treatment strategies for pneumococcal pneumonia are urgently needed. However, the long-term use of antibiotics, the current mainstay of treatment, easily leads to increased bacterial resistance, diminished therapeutic efficacy, and potential disruption of the normal human flora (Li et al., 2016).

The pathogenesis of *S. pneumoniae* involves transmission, colonization, and invasion. Pneumolysin (PLY) is a crucial virulence factor that participates throughout this pathogenic process (Weiser et al., 2018). PLY expression increases the *in vitro* survival of *S. pneumoniae* (Zafar et al., 2017). Upon invading the human body, PLY release reduces ciliary movement in respiratory epithelial cells, hinders bacterial clearance by cilia, and facilitates the endocytosis of *S. pneumoniae* into epithelial cells. PLY also directly damages the epithelial barrier. When *S. pneumoniae* breaches the endothelium and enters the bloodstream, PLY helps it effectively evade phagocytosis (Weiser et al., 2018). PLY consists of four domains, and at room temperature, many PLY monomers can form spiral oligomers. This oligomeric form exhibits a curved protein conformation, similar to a pore state, which then inserts into the cell membrane, causing cytotoxicity.

Currently, the main methods for preventing pneumococcal pneumonia involve the injection of pneumococcal polysaccharide vaccines and pneumococcal polysaccharide conjugate vaccines (Dinleyici, 2010), but antibiotic use remains the primary treatment. Although supplementary and alternative therapies such as natural compounds (Aelenei et al., 2016), nanotechnology (Park et al., 2016; Gunasekaran et al., 2019; Mirza et al., 2019), bacteriophages (Pastagia et al., 2013), antimicrobial peptides (Wang et al., 2019), and probiotics (Racedo et al., 2006; Villena et al., 2008; Salva et al., 2010) have been reported, none have achieved widespread clinical application. The existing methods have significant limitations: antibiotics are plagued by severe resistance-related issues (Campanini-Salinas et al., 2018); vaccines can accelerate the evolution of noninvasive serotypes (Straume et al., 2015); the specific therapeutic mechanisms of natural compounds remain unclear (Chandra et al., 2017); the safety of nanotechnology lacks a unified evaluation standard (Park et al., 2016; Gunasekaran et al., 2019; Mirza et al., 2019); bacteriophages and antimicrobial peptides are difficult to produce (Joerger, 2003); and the indirect bacteriostatic mechanisms of probiotics make direct evaluation of their therapeutic effects challenging (Markowiak and Śliżewska, 2017). Given these hurdles, further research for the development of novel drugs or vaccines is urgently needed to treat or prevent pneumococcal diseases (Dear et al., 2003; Fedson et al., 2004; Huss et al., 2009).

Traditional Chinese medicine (TCM) theory offers a unique perspective on diseases caused by pathogenic microorganisms. TCM views *S. pneumoniae* as an “external toxin” and emphasizes that the relationship between *S. pneumoniae* and the human body is not one of absolute antagonism. Instead, it posits that relatively balanced coexistence can be achieved through internal conditioning. From a TCM perspective, the pathogenic mechanism of *S. pneumoniae* involves invading the human body, damaging the lungs, and obstructing the flow of qi and blood. In treating such diseases, TCM adheres to the principle of “strengthening the body’s health and eliminating evil.” By regulating the functions of internal organs, TCM aims to bolster the body’s vital energy, enabling it to resist pneumococcal invasion. This approach does not seek to eliminate bacteria completely but rather aims to achieve harmonious coexistence between the human body and bacteria. This philosophy aligns with the holistic and equilibrium-based concepts of TCM, which emphasize that the human body is an organic whole and that disease arises from an imbalance between the body and its external environment. Presumably, restoring the body’s balance is key to the coexistence of pathogenic factors.

Among the many traditional Chinese medicines, Radix Paeoniae Alba (RPA) is particularly noteworthy. Traditionally, TCM posits that RPA prevents the excessive dissipation of body fluids and is primarily used to regulate the body in the later stages of pneumonia. Modern studies have shown that RPA can inhibit the release of inflammatory mediators and reduce intestinal damage (Li et al., 2022a), has antioxidant effects, scavenges free radicals, and protects the liver (Chen et al., 2023). Furthermore, it participates in the regulation of immune and inflammatory processes by modulating cell signaling pathways such as the NF-κB pathway (Wang et al., 2020). These mechanisms are inextricably linked to inflammation. Given the role of RPA in regulating body fluids according to TCM and its demonstrated modern pharmacological actions related to inflammation and immune modulation, it appears to have significant potential for further development in the treatment of *S. pneumoniae*-related diseases.

In our research, we aimed to apply the TCM concept of “balance” to investigate the efficacy of RPA, focusing on its ability to inhibit the toxicity of *S. pneumoniae* rather than directly inhibiting its growth. We endeavor to provide novel insights for the continued development of RPA.

## 2 Materials and methods

### 2.1 Cells, bacteria, and animals

A549 alveolar epithelial cells were purchased from ATCC (Manassas, VA, United States) and cultured in DMEM (Gibco Life Technologies, Inc., Grand Island, NY, United States) supplemented with 10% fetal bovine serum (FBS; Biological Industries, Israel). *S. pneumoniae* D39 serotype 2 (NCTC7466) (a gift from Professor Huang Jian, Zunyi Medical University) was cultured in THY medium at 37 °C under 5% CO2 in an incubator.

A total of 50 BALB/c mice (female, 6–8 weeks old, 20–22 g) were purchased from Liaoning Changsheng Biotechnology Co., Ltd. (Laboratory Animal Production License No. SCXK (Liao) 2020–0001) and were housed at a temperature of 24 °C ± 3 °C, a relative humidity of 40% ± 5%, a 12-h light/12-h dark cycle, a noise level <55 Db, and free access to water and food; the litter was changed twice a week. The animal experiments were approved by the Experimental Animal Welfare Ethics Committee of Henan University of Chinese Medicine (IACUC-202404012).

### 2.2 PLY and hemolysis assays

PLY was purchased from Fitzgerald (80R-4390, United States). Different concentrations of white peony root (0, 4, 8, 16, 32, or 64 μg/mL) were mixed with PLY and incubated in PBS at 37 °C for 30 min (Tilley et al., 2005; Guo et al., 2021; Parveen et al., 2024; Vorobyev et al., 2024). Then, 25 μL of defibrinated sheep red blood cells was added to the mixture and incubated at 37 °C for 10 min. Finally, the mixture was centrifuged (Eppendorf 5424R, Eppendorf AG, Hamburg, Germany) at 3,000 × g for 5 min, and the resulting supernatant was collected to measure the hemolytic activity by recording the OD at 543 nm using a microplate reader (Tecan Infinite M200 Pro, Tecan Austria GmbH, Grödig, Austria). The OD value at 543 nm was used to determine the concentration of hemoglobin released after red blood cell lysis, thus serving as a measure of hemolytic activity.

### 2.3 Antibacterial activity of RPA and inhibition curve analysis

White peony root (*Radix Paeoniae Alba*, or RPA) was purchased from Jiangyin Tianjiang Pharmaceutical Co., Ltd. (product lot number: 21,060,071). RPA is the dried root of *Paeonia lactiflora Pall*., which is a member of the Ranunculaceae plant family. For preparation, 4,500 g of prepared RPA slices were first decocted with water. The decoction was subsequently filtered, and the resulting filtrate was concentrated into a clear paste (a concentrated extract), with a dry extract yield of 14–22% being achieved. Finally, by the addition of appropriate excipients and implementing processes such as drying and granulation, the clear paste was processed into a convenient granular form. The preparation standard for this process followed the Chinese National Standard YBZ-PFKL-2021002. The sample was subsequently dissolved in pure DMSO at 100 mg/mL and stored at −20 °C as a stock solution.

The concentrations of RPA used for the *in vitro* assays (4, 8, 16, 32, and 64 μg/mL) were selected on the basis of a series of preliminary experiments. The minimum inhibitory concentration of RPA was determined by the microbroth method, and different concentrations of RPA (0, 4, 8, 16, 32, and 64 μg/mL) were cultured with *S. pneumoniae* in Todd-Hewitt broth supplemented with 1% yeast extract (THB). The growth of *S. pneumoniae* was monitored every 30 min at 600 nm using a UV spectrophotometer. In each well of a 96-well plate, 2 × 10^4^ A549 cells were added and incubated overnight, 3.0 μL of PLY pretreated with different concentrations of RPA (0, 4, 8, 16, 32, or 64 μg/mL) was added to each well, and these samples were placed in an incubator at 37 °C and cultured for 5 h. The cells were treated with a live/dead (green/red) staining kit (Invitrogen, Carlsbad, CA, United States) according to the manufacturer’s instructions, and cell viability was determined via a confocal laser scanning microscope (FluoView FV3000, Olympus, Tokyo, Japan).

### 2.4 Western blot detection

*S. pneumoniae* D39 was cultured in THY, and RPA (0, 4, 8, 16, 32, or 64 μg/mL) was added at 37 °C. After centrifugation (3,000 rpm, 10 min), 5× SDS‒PAGE solution (10% SDS, 50% glycerol, 5% β-mercaptoethanol, 0.05% bromophenol blue, 0.3125 M Tris-HCl, pH 6.8) was added, and the mixture was incubated at 100 °C for 10 min. Samples were separated on 12% SDS‒PAGE gels and transferred to a PVDF membrane, which was then blocked with 5% skim milk powder at room temperature for 2 h.

The membrane was incubated with an anti-PLY monoclonal antibody (ab71810, 1:1,000; Abcam, Cambridge, United Kingdom) at 4 °C overnight, washed with PBST, and incubated with a secondary antibody (1:2000; Proteintech, Chicago, IL, United States) at 37 °C for 1 h. After washing with PBST, the membrane was developed using a Tanon-4200 imager (Tanon, Shanghai, China) and enhanced chemiluminescence (ECL) reagent (Thermo Scientific, Rockford, IL, United States).

The methods used for oligomer and monomer detection were the same as those described above: different concentrations of RPA and PLY were incubated at 37 °C for 1 h and then boiled at 50 °C for 10 min (5× SDS‒PAGE loading buffer without β-mercaptoethanol was added). Then, oligomers and monomers of PLY were visualized and analyzed.

### 2.5 Mouse model of pneumococcal infection

Pneumococcal D39 cells were cultured in THY medium at 37 °C to the mid-logarithmic phase (OD600 nm = 0.4), washed three times with PBS via centrifugation, and resuspended in PBS. The 50 mice were evenly divided into 5 groups: (1) the Control group (uninfected, treated with PBS); (2) the D39+PBS group (infected, treated with PBS); (3) the D39 + 25mgRPA group (infected, treated with 25 mg/kg RPA); (4) the D39 + 50mgRPA group (infected, treated with 50 mg/kg RPA); and (5) the 50mgRPA + PBS group (uninfected, treated with 50 mg/kg RPA as a drug toxicity control).

All of the mice were lightly anesthetized with ether. For the D39+PBS, D39 + 25mgRPA, and D39 + 50mgRPA groups, 1.5 × 10^8 colony-forming units (CFUs) were inoculated into the left nostril for lung infection (Guo et al., 2021). For the control group, an equivalent volume of PBS was intranasally instilled as a vehicle control. All of the groups subsequently received subcutaneous injections; specifically, the D39 + 25mgRPA group received 25 mg/kg RPA every 8 h, while the D39 + 50mgRPA and 50mgRPA + PBS groups received 50 mg/kg RPA every 8 h. The D39+PBS and Control groups received an equivalent volume of PBS every 8 h as a vehicle control. The dosage of RPA used in this study (50 mg/kg) was determined on the basis of the results of preliminary dose range experiments. The dose range for these preliminary experiments was established on the basis of the conventional clinical human dose of RPA, with the corresponding mouse equivalent dose being estimated according to the standard pharmacological principle of body surface area (BSA) conversion.

The survival of each mouse was recorded, and the remaining mice were euthanized 48 h after infection. Bronchoalveolar lavage fluid (BALF) was collected from the mice. The collected BALF was initially centrifuged, after which the supernatant was used for subsequent analysis. The levels of cytokines (IL-1β (BMS6002-2), IL-6 (BMS603-2), and TNF-α (88–7,324-88)) in the supernatant were determined by ELISA kits (Thermo Fisher Scientific (eBioscience), San Diego, CA, United States). The dry and wet weights of the lung tissues were measured, and the wet weight/dry weight ratio was calculated.

Bronchoalveolar Lavage Fluid Collection: After euthanasia, the mice were placed in the supine position on the operating table. The chest cavity was carefully opened to expose the trachea, and a small incision was made. A blunt-ended needle was then inserted into the trachea and secured with a suture. PBS was slowly infused into the lungs, with each infusion consisting of 0.5–1 mL. Each mouse’s chest was gently massaged to ensure thorough mixing of the lavage fluid within the alveoli. The lavage fluid was subsequently withdrawn, and this process was repeated 2 to 3 times, recovering approximately 1.0–2.4 mL of BALF (70%–80% recovery rate).

Macroscopic and Histological Assessments of Lung Tissues: After the mice were euthanized, the lung tissues were carefully excised and rinsed with precooled phosphate-buffered saline (PBS). For macroscopic assessments, the intact lungs were placed onto a white background and immediately photographed. Macroscopic pathological changes in the lung tissues, including congestion, edema, and consolidation, were visually observed and recorded. To objectively quantify the degree of pulmonary edema, the wet/dry weight ratio of the left lung tissue was determined. Specifically, the left lung was isolated and immediately weighed to obtain its wet weight. The same lung tissue was subsequently dried in an oven at 70 °C for 72 h until a constant dry weight was achieved, after which the dry weight was measured. Finally, the ratio of the wet weight to dry weight (wet/dry weight ratio) was calculated to reflect the degree of pulmonary edema. For histological assessments, lung tissue samples were immediately fixed in 10% neutral buffered formalin solution for at least 24 h. After fixation, the tissues were sequentially dehydrated (via gradient ethanol), cleared (via xylene), and embedded in paraffin. Continuous sections (3–5 micrometers thick) were subsequently cut by using a paraffin microtome. The sections were mounted on glass slides and stained with hematoxylin‒eosin (H&E). Stained lung tissue sections were observed under an optical microscope, and representative images were captured. The main observations included observation of whether the alveolar lumens were filled with inflammatory cells and exudates, the extent of inflammatory cell infiltration and the overall morphology of the alveolar structures.

### 2.6 Statistical methods

Statistical analyses were performed using GraphPad Prism 8 software. One-way analysis of variance (ANOVA) followed by Tukey’s post hoc test was used for multiple group comparisons. The data are presented as the means ± standard deviations (means±SDs). A p value <0.05 was considered to indicate statistical significance.

## 3 Results

### 3.1 RPA reduces lung inflammation in mice infected with *S. pneumoniae*

**FIGURE 1:**
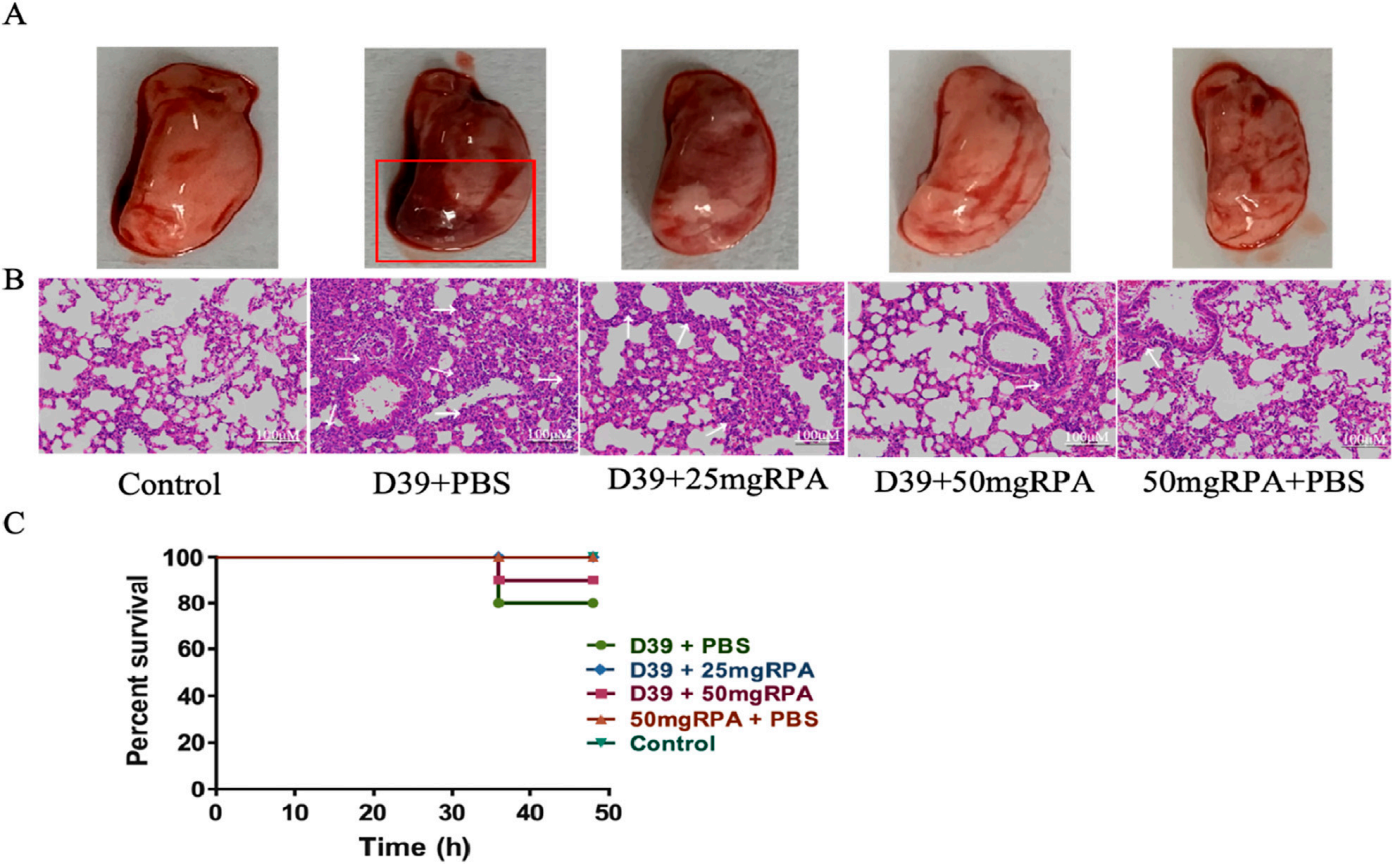
RPA reduces lung inflammation in mice infected with S. pneumoniae. **(A)** Macroscopic view of the lungs from all five experimental groups. The red box highlights severe pulmonary congestion and consolidation in the lung tissue from the D39+PBS group. **(B)** Pathological views of the lungs from the different groups. **(C)** Survival rate of the mice at 48 h, showing the results from all five groups. The arrows indicate severe inflammatory responses in the lung tissue, where alveolar lumens are extensively filled with inflammatory cells and exudates, leading to the loss of normal alveolar structure and widespread inflammatory cell infiltration. The initial number of mice in each group was n = 10, and survival curves were analyzed using the log-rank (Mantel‒Cox) test. The results shown in the figure are representative findings.

We established a mouse model of *S. pneumoniae* infection to explore the effect of RPA administration on *S. pneumoniae* infection *in vivo*. Compared with the uninfected healthy control group, the *S. pneumoniae* model group presented obvious symptoms of lung infection, including lung tissue congestion, consolidation, and a severe inflammatory response (Figure 1A).

After RPA treatment at both 25 mg/kg and 50 mg/kg, lung infection symptoms in the mice were significantly reduced, and the degree of congestion and consolidation in the lung tissues was significantly relieved, with significant differences observed compared to the model group. Further pathological examinations of the mouse lungs revealed that the lung tissue of the D39+PBS model group exhibited a severe inflammatory response, with alveolar lumens being extensively filled with inflammatory cells and exudates, thereby leading to blurred normal alveolar structures and widespread inflammatory cell infiltration (Figure 1B). In contrast, the lung pathology of the mice treated with both 25 mg/kg and 50 mg/kg of RPA was significantly improved, with the 50 mg/kg group exhibiting a more normal appearance, alveolar lumens reopening, significantly reduced inflammatory cell infiltration, and good recovery of lung tissue structures (Figure 1B). The lung pathology of the RPA-treated mice was significantly improved, with a relatively intact alveolar structure, reduced inflammatory cell infiltration, and decreased alveolar septal thickness (Figure 1B), which corresponded to a higher survival rate (Figure 1C). These results indicate that RPA administration can effectively reduce lung inflammation caused by *S. pneumoniae*.

### 3.2 RPA administration reduces the level of inflammatory factors in the lungs of mice infected with *S. pneumoniae*

**FIGURE 2:**
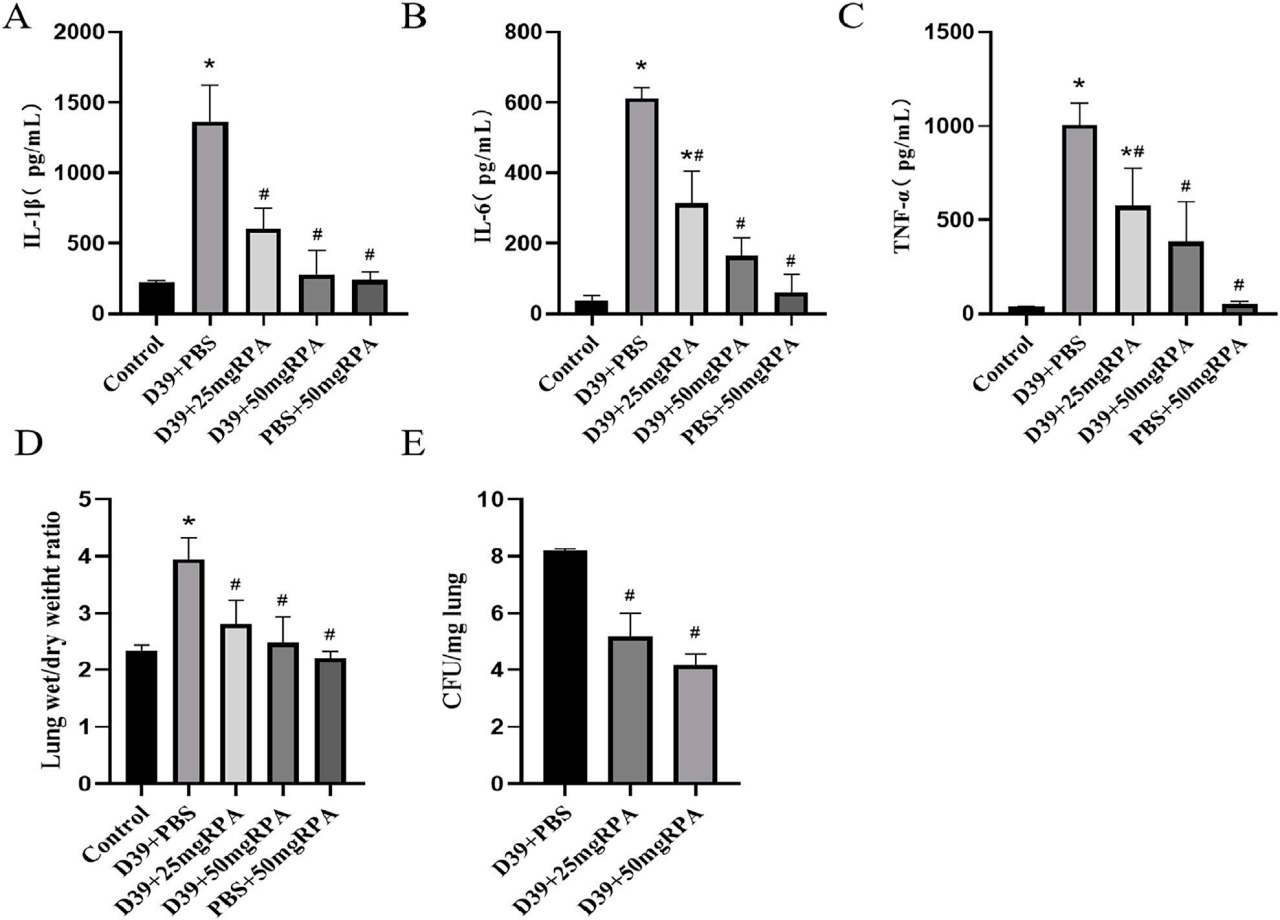
RPA administration reduces the levels of inflammatory factors in the lungs of mice infected with *S. pneumoniae*. **(A)** The expression of IL-1β in each group; **(B)** the expression of IL-6 in each group; **(C)** the expression of TNF-α in each group; **(D)** the wet/dry ratio of the lung tissues in each group; and **(E)** the bacterial abundance in the lung tissues in each group. n ≥ 3, * represents a comparison with the control group, *P* < 0.05; # represents a comparison with the D39+PBS group, *P* < 0.05. Statistical significance was determined using one-way ANOVA with Tukey’s post hoc test.

The levels of the inflammatory factors IL-1β, IL-6 and TNF-α in the BALF of the mice in the model group were significantly increased, whereas after treatment with both 25 mg/kg and 50 mg/kg of RPA, the levels of IL-1β, IL-6 and TNF-α were significantly decreased (Figures 2A–C). These findings confirm that RPA administration can effectively reduce the pulmonary inflammatory response caused by *S. pneumoniae*. Moreover, the wet/dry ratio of the lung tissues increased significantly after *S. pneumoniae* infection, whereas administration of both RPA doses reduced the wet/dry ratio of the lung tissues (Figure 2D). Similarly, the number of *S. pneumoniae* in the lungs was significantly reduced after intervention with both doses of RPA (Figure 2E). For all these indicators, a greater therapeutic effect was observed in the 50 mg/kg group compared to the 25 mg/kg group.

### 3.3 RPA administration reduces the effect of PLY on A549 cell death

**FIGURE 3:**
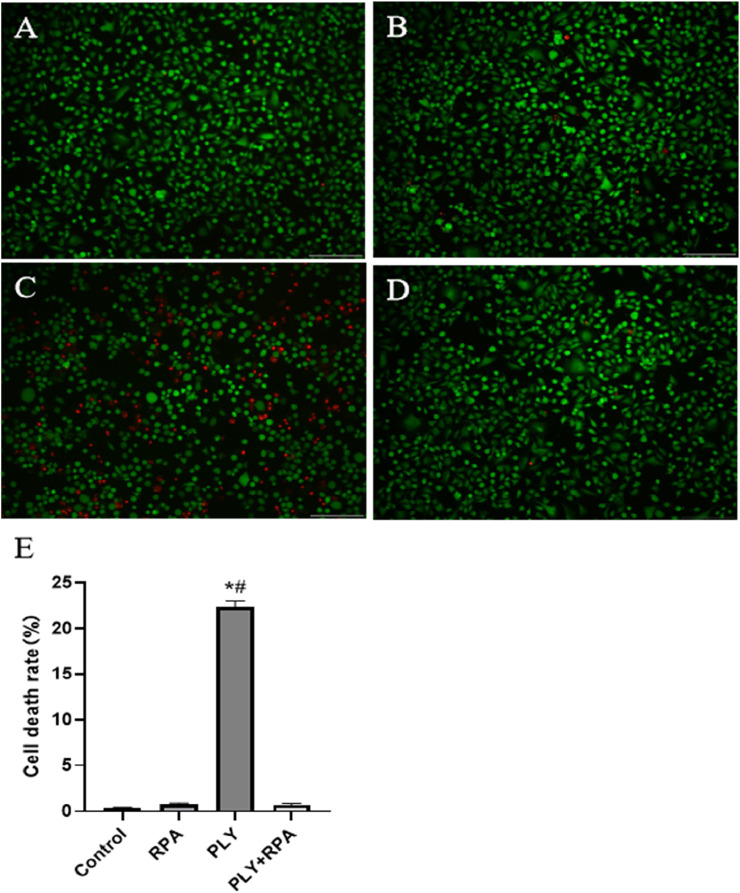
RPA administration reduces the PLY-mediated death of A549 cells. **(A)** Untreated cells, **(B)** cells treated with 64 μg/mL RPA, **(C)** cells treated with PLY, and **(D)** cells treated with 64 μg/mL RPA and PLY. **(E)** Quantitative analysis of the cell death rates in the different treatment groups. The data are presented as the means ± SDs from three independent experiments. Scale bars: 150 µm *P < 0.05 compared with the control group; #*P* < 0.05 compared with the PLY + RPA group. The statistical analysis was performed using one-way ANOVA followed by Tukey’s post hoc test.

The invasion process of *S. pneumoniae* is very complex, and PLY plays a major role and is a major virulence factor. In the A549 cytotoxicity experiment, we found that the 64 μg/mL RPA solution had no obvious effect on A549 cells, and the cell death rate was directly observed via microscopy. The results revealed that when RPA was not added to PLY, large numbers of A549 cells died; however, after preincubation with 64 μg/mL RPA with PLY and subsequent addition of the mixture to A549 cells, the number of dead cells in the visual field was significantly reduced (Figure 3). These results indicate that RPA administration can inhibit PLY-mediated cell death, thereby reducing its ability to damage cells.

### 3.4 RPA administration does not affect the growth of *S. pneumoniae* but can reduce the PLY-induced hemolysis of erythrocytes

**FIGURE 4:**
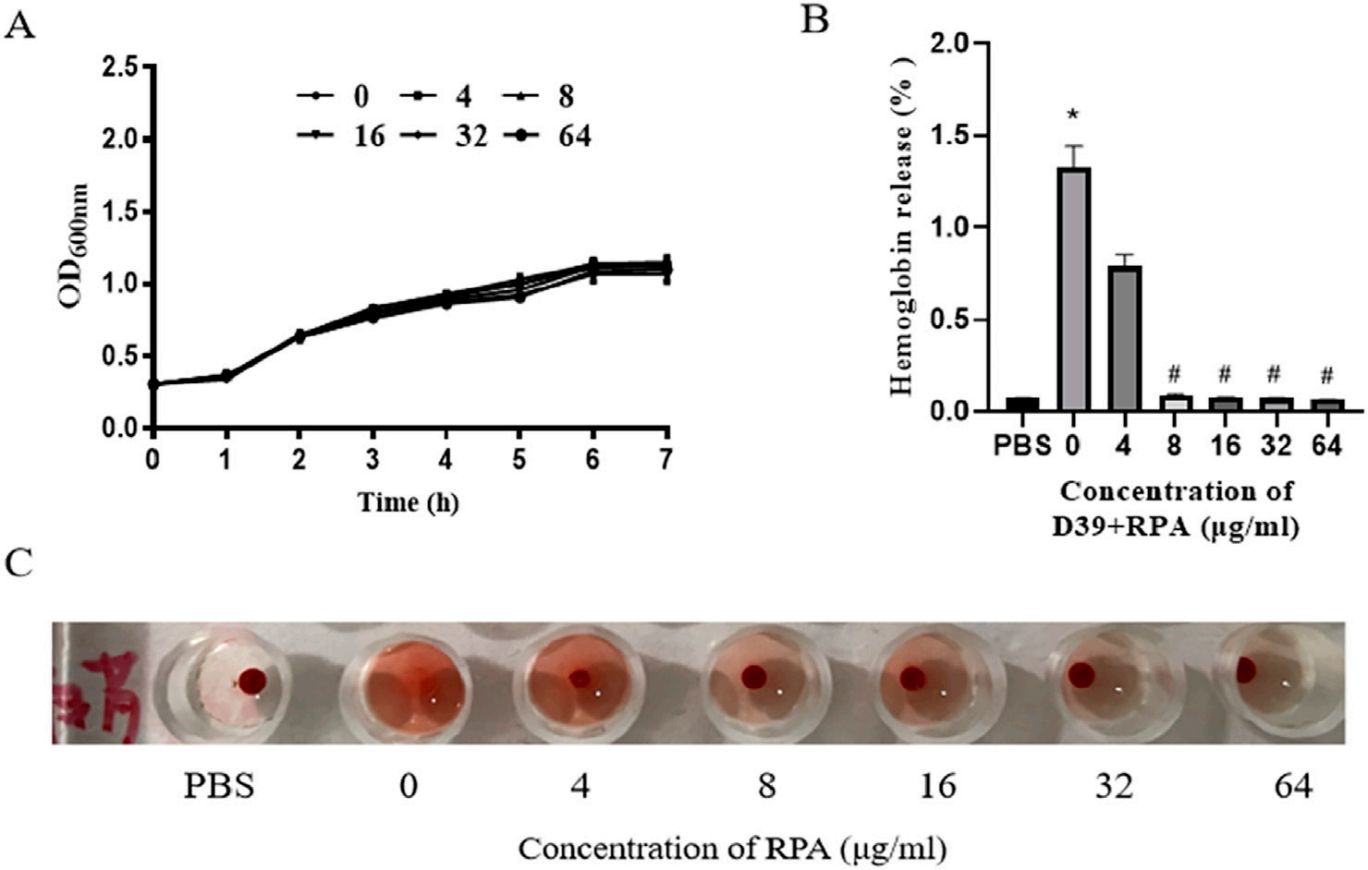
RPA administration does not affect the growth of *S. pneumoniae* but can reduce the PLY-induced hemolysis of erythrocytes. **(A)** Effects of different concentrations of RPA on the growth of *S. pneumoniae*. **(B)** Effects of different concentrations of RPA on the hemolysis of sheep red blood cells. **(C)** Macroscopic observation of the effects of different concentrations of RPA on hemolysis. The data are presented as the means ± SDs from three independent experiments. *P < 0.05 compared with the PBS group; #*P* < 0.05 compared with the 0 μg/mL group. Statistical significance was determined using one-way ANOVA followed by Tukey’s post hoc test.

We next tested the inhibitory effect of RPA on *S. pneumoniae*. As shown in Figure 4A, we found that RPA administration may not inhibit or reduce the toxicity of *S. pneumoniae* by directly inhibiting its growth. We also tested the effect of RPA administration on the PLY-mediated hemolytic activity toward sheep red blood cells. The results showed that RPA administration clearly inhibited the PLY-mediated hemolysis of red blood cells in a concentration-dependent manner (Figure 4C).

### 3.5 RPA administration reduces PLY oligomerization

**FIGURE 5:**
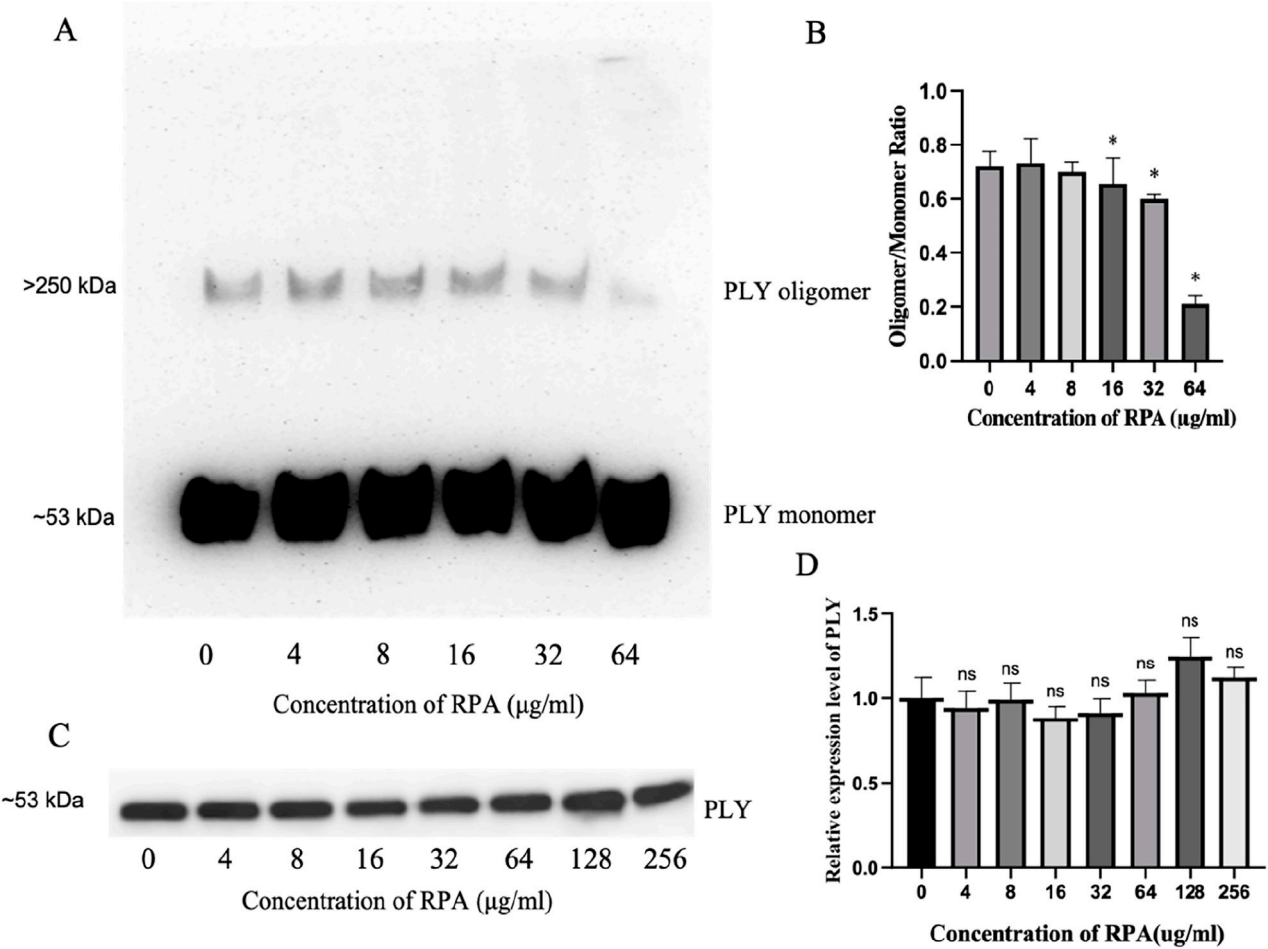
RPA inhibits PLY oligomerization without affecting PLY production by *S. pneumonia*e. **(A)** A representative Western blot showing the dose-dependent inhibitory effect of RPA on PLY oligomerization. Molecular weight markers indicate the positions of the PLY monomer (∼53 kDa) and oligomer complexes (>250 kDa). **(B)** Densitometric analysis of the oligomer-to-monomer ratio from three independent experiments (n = 3). **(C)** A representative Western blot showing the effects of different concentrations of RPA on PLY production in *S. pneumoniae* culture supernatants. The molecular weight marker indicates the position of the PLY monomer (∼53 kDa). **(D)** Densitometric analysis of PLY production from three independent experiments (n = 3). The data are presented as the means ± SDs. *P < 0.05 compared with the 0 μg/mL group. ns indicates no significant difference compared with the 0 μg/mL group. Statistical significance was determined using one-way ANOVA with Tukey’s post hoc test.

These results clearly show that RPA administration reduces lung inflammation after pneumococcal infection by targeting PLY. PLY consists of four domains that form a complex spatial structure. Many PLY monomers form helical oligomers at room temperature. The oligomer presents a curved protein conformation similar to that of a pore state. This special structure enables PLY to insert into the host cell membrane, form a transmembrane channel, and cause leakage of intracellular substances, ultimately causing cell death. Therefore, we tested whether RPA administration reduces the toxicity of pneumococcal pneumonia by reducing the oligomerization of PLY, as shown in Figure 5. As shown by the Western blot data in Figure 5A, RPA inhibited the formation of PLY oligomers in a dose-dependent manner. Quantitative densitometric analysis of three independent experiments confirmed this observation, revealing a statistically significant reduction in the oligomer-to-monomer ratio at concentrations of 16 μg/mL and higher (Figure 5B).

Next, we investigated whether RPA affects the production of PLY by *S. pneumoniae* and found that RPA did not significantly affect the production of PLY, as shown by the representative blot in Figure 5C. This finding was further confirmed by quantitative analysis, which revealed no statistically significant differences in band intensity across all the tested concentrations (Figure 5D).

Therefore, the above results show that RPA administration reduces the toxicity of pneumococcal pneumonia by directly inhibiting the oligomerization of its key virulence factor, PLY, rather than by affecting its production.

## 4 Discussion

### 4.1 Antivirulence mechanism of RPA in *S. pneumoniae* infection

This study revealed that RPA can specifically inhibit the oligomerization of pneumolysin (PLY), which is a crucial virulence factor of *Streptococcus pneumoniae*, thereby significantly reducing the toxicity of *S. pneumoniae*. Our Western blot results clearly demonstrated that RPA effectively reduced the formation of PLY oligomers in a concentration-dependent manner (with a significant effect being observed at 64 μg/mL). As a key virulence factor of *S. pneumoniae*, PLY requires oligomerization into a transmembrane pore structure to exert hemolytic and cytotoxic effects, thereby promoting bacterial invasion and damaging host cells. Therefore, the inhibition of PLY oligomerization is a key strategy to block its pathogenicity. *In vitro* hemolysis assays and A549 cell cytotoxicity experiments further validated the inhibitory effect of RPA on PLY activity, thus demonstrating that RPA can effectively inhibit PLY-mediated erythrocyte hemolysis and alveolar epithelial cell damage.

In the *in vivo* infection model, the therapeutic effect of RPA was equally significant; specifically, it not only significantly alleviated lung inflammation in *S. pneumoniae*-infected mice (as evidenced by reduced lung tissue congestion and consolidation) and improved histopathological results but also decreased the release of inflammatory factors (such as IL-1β, IL-6, and TNF-α) and significantly improved pulmonary edema (as reflected by a decrease in the wet/dry weight ratio). Furthermore, we observed a reduction in *S. pneumoniae* bacterial counts in the lungs after RPA intervention, suggesting that RPA may indirectly affect the pathogen load, thus facilitating clearance from the host. These *in vitro* and *in vivo* findings collectively and comprehensively delineate the efficacy and mechanism of the antivirulence effects of RPA in *S. pneumoniae* infection.

### 4.2 Unique advantages and potential of RPA in combating antibiotic resistance

As a multicomponent traditional Chinese medicine, RPA has distinct advantages and considerable potential compared with conventional single-target drugs when the increasingly severe issue of antibiotic resistance is considered. Antibiotic resistance has become one of the most important threats to global public health, leading to hundreds of thousands of deaths annually and projected to cause tens of millions of deaths by 2050 (Subramaniam and Girish, 2020). The primary mechanism of action for conventional anti-infective drugs (particularly antibiotics) is to kill or inhibit bacterial growth. However, although this strategy has resulted in immense benefits to humans, it has also imposed tremendous evolutionary selective pressure on bacteria, thereby leading these organisms to develop resistance mechanisms such as reduce antibiotic uptake, alteration of targets, or modification of the antibiotic itself (Li et al., 2022b; Zhou et al., 2022), as well as rapid dissemination of resistance genes via horizontal gene transfer (HGT) (McInnes et al., 2020). The widespread misuse of antibiotics (including their extensive use in livestock farming) has resulted in their broad presence in water bodies, soil (Rocha et al., 2021), and the food chain, thereby further exacerbating the global antibiotic resistance crisis (Zhang et al., 2015). In this context, there is an urgent need for the international research community to develop novel anti-infective strategies (Choi et al., 2019; Liu et al., 2018; Li et al., 2020).

The antivirulence mechanism of RPA fundamentally differs from that of conventional antibiotics. This study revealed that RPA alleviates toxicity by targeting PLY oligomerization; however, it does not directly inhibit the growth of *S. pneumoniae* or affect the production of PLY. This finding indicates that RPA does not exert direct selective pressure on bacterial survival and reproduction (Abdal et al., 2019). This “nonbactericidal” antivirulence strategy may effectively circumvent the main drivers of bacterial resistance development, thus reducing the risk of resistant strain emergence. This scenario represents a burgeoning area of interest in anti-infective drug research and development, thereby aiming to “disarm” (rather than eradicate) pathogens and enable the host immune system to more effectively clear infections.

The multicomponent nature of RPA also offers potential advantages beyond single-target drugs. Unlike single-component drugs, which may quickly lose efficacy because of bacterial target mutations, multicomponent TCMs can theoretically act on multiple pathogen targets or host physiological pathways, thereby providing broader or more sustained therapeutic effects and reducing the likelihood of the emergence of single resistance mechanisms. For example, our research group has explored how other TCM formulas, such as Ma-xing-shi-gan-tang (MXSGT) (Guo et al., 2021), inhibit PLY oligomerization to exert antivirulence effects against *S. pneumoniae* without affecting bacterial growth or PLY production. Furthermore, active components isolated from licorice, such as glycyrrhetinic acid and glycyrol, have been confirmed to reduce *S. pneumoniae* toxicity by inhibiting PLY oligomerization without affecting bacterial growth or PLY production, thus further supporting the antivirulence potential of TCMs (Li et al., 2024; Xu et al., 2024). These studies collectively reveal the broad prospects of TCMs in targeting bacterial virulence factors.

Moreover, TCMs benefit from thousands of years of clinical use, and their safety has been widely validated. Some studies have indicated that TCM components can increase bacterial sensitivity to antibiotics, thereby improving antibiotic treatment efficacy (Zhou et al., 2022). Additionally, TCMs can further reduce the necessary dosage and side effects of antibiotics by adjusting human immune function and enhancing the body’s absorption and utilization of antibiotics, thus offering new avenues for combination therapies. These characteristics make natural products (especially TCMs, which have a long history of use) highly attractive for the identification of novel anti-infective drugs.

**FIGURE 6:**
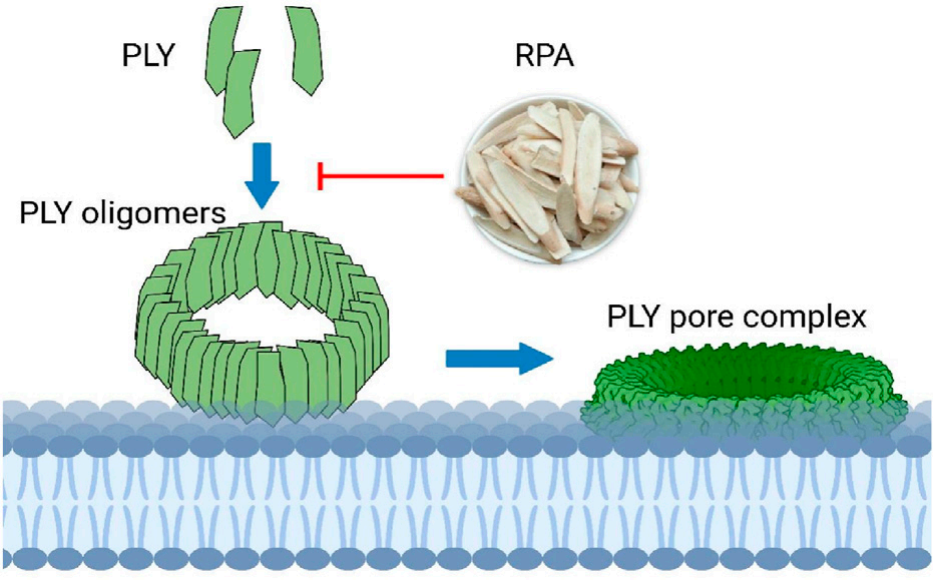
RPA targets PLY oligomerization to reduce its pore-forming activity.

This study clearly demonstrated that RPA can inhibit the virulence of *S. pneumoniae* without imposing evolutionary pressure, as shown in Figure 6. This finding aligns with the holistic concept of TCM and its “body-strengthening and evil-eliminating” (扶正祛邪) therapeutic principle. The use of TCM is based on the belief that disease arises from insufficient zhengqi (the body’s vital energy) and the invasion of xieqi (pathogenic factors), thus leading to an imbalance in the body. In TCM, RPA is considered to have the ability to nourish yin-fluid and harmonize ying-wei (nutritive and protective qi), which is consistent with the observed effects of RPA in alleviating inflammation and restoring pulmonary tissue homeostasis (Wu et al., 2023). By inhibiting the virulence factors of the pathogen, RPA weakens its pathogenicity, thereby creating conditions for the body’s immune system to restore its own balance and clear the infection. This scenario precisely embodies the harmonious unity of “strengthening the body” (扶正) and “eliminating evil” (祛邪). This moderate intervention may also help maintain the stability of the host microbiota, thereby avoiding the dysbiosis that is often associated with broad-spectrum antibiotics.

### 4.3 Study limitations and future directions

Despite the demonstrated antivirulence potential of RPA, this study has several limitations. First, the tested concentration range of RPA may not be sufficient to completely inhibit PLY oligomerization. Future studies should explore whether higher concentrations of RPA can further reduce the levels of residual oligomers. Second, the results from the *in vitro* experiments may not fully reflect the role of RPA in the complex *in vivo* environment. Therefore, future research should validate these findings via clinical trials. Furthermore, the *in vivo* experimental design, while appropriate for a proof-of-concept study, has several limitations. The use of a single effective dose and a sample size of n = 10 per group was sufficient for the primary pathological endpoints at 48 h but was not intended for a full dose‒response or long-term survival analysis. Consequently, the survival curve should be interpreted as an observation of the acute phase. Additionally, a traditional antibiotic was not included as a positive control, as the primary goal was to validate the novel antivirulence mechanism of RPA, which differs fundamentally from that of bactericidal agents. Additionally, residual oligomers detected via Western blot analysis may still retain some biological activity, which could impact the therapeutic efficacy of RPA. Future studies should assess the activity of these residual oligomers and their influence on the overall treatment outcome.

In conclusion, this study preliminarily demonstrated the innovative mechanism by which TCM RPA effectively reduces *S. pneumoniae* toxicity by the specific inhibition of PLY oligomerization. This study provides a scientific basis for the development of novel, less resistance-inducing anti-infective strategies and offers a new paradigm for the integration of modern medicine and TCM. The antivirulence action of RPA, along with its “no evolutionary pressure” characteristic, positions it as a potential breakthrough in addressing the global antibiotic resistance crisis, thereby offering insights for the development of smarter and more sustainable anti-infective strategies. Future research directions include (but are not limited to) the following: further elucidation of the active components within RPA that are responsible for inhibiting PLY oligomerization; assessment of the broad-spectrum activity of RPA across different *S. pneumoniae* serotypes and clinical isolates; exploration of the synergistic effects of RPA in combination with other antibiotics or antivirulence agents; and thorough investigation of the impact of RPA on host immune modulation and microbiota balance, thereby realizing a more comprehensive “body-strengthening and evil-eliminating” anti-infective strategy.

## Data Availability

The original contributions presented in the study are included in the article/supplementary material, further inquiries can be directed to the corresponding author.
